# View of a London Secretary

**Published:** 1914-09-12

**Authors:** 


### HOSPITALS AND THEIR CONTRACTS.*

### View of a London Secretary.

The boards of management of the voluntary hospitals, in administering the funds entrusted to them by the supporters of their institutions, have at all times to remember that they are acting as trustees and cannot therefore be as generous as if they were dispersing their own private funds. Those who have elected them, however, will no doubt expect them to be just at all times, if they cannot afford to be generous, and it is in such a spirit that the difficulties arising out of contracts must be faced.

These contracts appear to me to divide themselves into two different classes; the first, including groceries of all kinds and non-perishable goods, should in prudence have been covered by the contractors by large stocks and forward purchases. The second, which includes butchers' meat and other perishable articles, are not so easily protected, and contractors dealing in these articles deserve more careful consideration than those in the former class.

In all these contracts the firms who come to their contractees and ask for consideration will no doubt receive more sympathy than those who take a truculent attitude. The latter are more difficult to deal with, and as an example of the case I mean I will quote from a letter received on August 4, but a few hours after the trouble commenced. It is from a large firm of grocers, who for many years have held the majority of the contracts of the London hospitals, as well as doing a large trade with Poor-Law and other institutions. They have a warehouse of considerable size, which at the commencement of the war must have been well stocked.

"We are compelled to inform you that all orders received from you from the beginning of this week and henceforth are only accepted on the understanding that they be executed at prevailing market prices. Quantities of very many articles are exceedingly short, but owing to our long business relations with you you will receive our earliest consideration. We shall, of course, invoice you at strictly market rates."

This firm immediately began to make short deliveries, and for at least a week would not answer letters, telegrams, or telephone messages. Early in the first week of the war they sent a contribution of £1,000 to the Prince of Wales' Relief Fund, which, it may not be unfair to say, was, at least partly, at the expense of their contractees. The answer in the case of the hospital I serve was that their letter would receive consideration in due course, but that I could only say for the present that our business relations were governed by the contract between us.

We are advised by our solicitors that the fact that war has broken out does not affect the liability of contractors, since this contingency is not specially provided for in the contract. The only cause which would excuse a discontinuance of supplies at the prices and on the terms of that document would be physical impossibility to carry it out; but at present there is no such impossibility, and therefore the hospital is entitled to hold the contractor to the strict terms of the contract. I do not think that any hospital is entitled to give in entirely on this point. It must necessarily be a question of give and take, each case being considered on its merits, and in view of the fact that most of the contractors have been doing business with London hospitals for a number of years, and presumably have made a fair profit out of them, always having the benefit of the fall in the price of goods, no bad debts having ever been made, it may be that the final settlement will be on the lines of the contractors and contractees sharing the loss between them.

* A Provincial Secretary's View appeared in The Hospital, August 29, p. 594.

